# Feeding practices in Australian early childhood education and care settings

**DOI:** 10.1017/S1368980021004055

**Published:** 2022-02

**Authors:** Rebecca A Byrne, Kimberley Baxter, Sue Irvine, Helen Vidgen, Danielle Gallegos, Elizabeth Martin, Stewart G Trost

**Affiliations:** 1 Queensland University of Technology (QUT), School of Exercise and Nutrition Sciences, Faculty of Health, Centre for Children’s Health Research (CCHR), Kelvin Grove, QLD 4101, Australia; 2 Queensland University of Technology (QUT), School of Early Childhood and Inclusive Education, Faculty of Creative Industries, Education and Social Justice, Kelvin Grove, QLD, Australia; 3 Queensland University of Technology (QUT), School of Public Health and Social Work, Faculty of Health, Kelvin Grove, QLD, Australia

**Keywords:** Early childhood education and care, Educators, Early childhood, Feeding practices, Children

## Abstract

**Objective:**

Feeding practices used by educators in Early Childhood Education and Care (ECEC) settings can influence the diet quality of young children. However, Australian data is scarce and limited to describing barriers to responsive feeding. This study describes the use of feeding practices amongst a group of Australian educators.

**Design:**

Direct observation of feeding practices and assessment of centre policy were conducted using the ‘Environment and Policy Assessment and Observation’ tool. Self-reported feeding practices and demographic data were collected via online survey using the Childcare Food and Activity Practices Questionnaire.

**Setting:**

Ten centre-based ECEC services in South East Queensland, Australia.

**Participants:**

Educators working in ECEC.

**Results:**

A total of 120 meals were observed and 88 educators provided self-report data (*n* 84 female). Centre policy supported the use of responsive feeding practices, and this was reflected in the high frequency with which children could decide what and how much to eat, across both observed and self-report data as well as low levels of pressure to eat and use of food as a reward (observed at 19·9 % and 0 % of meals). The only apparent discrepancy was regarding modelling. Median score for self-reported role-modelling was 5·0 (4·3–5·0) and educators were observed to sit with children at 75 % of meals, however observed occasions of enthusiastic role modelling was only 22 % (0–33·3) of meals.

**Conclusions:**

Research addressing how educators conceptualise feeding practices, as well under what circumstances they are used, particularly in centres with different models of food provision, may shed light on why modelling is rarely implemented in practice.

Birth to 5 years of age is a key window for children to learn about food and eating and the importance of optimal diet quality and mealtime environments in early childhood cannot be underestimated. The variety and type of food young children are exposed to, and opportunities to observe and imitate others’ eating behavior can influence life-long food preferences and growth trajectory^(1)^. Children are born with the ability to self-regulate their energy intake^(2)^, highlighting the importance of providing children with nutritious food and allowing them to follow internal cues of hunger and satiety.

How young children are fed by adults can support or undermine self-regulation and research into the impact of parental feeding practices on child outcomes is well-established^(3,4)^. ‘Feeding practices’ are the methods parents (and other carers) use at mealtimes to ensure children eat the amount and type of food that the caregiver deems is appropriate; and are broadly classified into three domains – autonomy support, structure and coercive control^(5)^. Within the domains of autonomy support and structure, ‘*prompt, contingent and developmentally appropriate responses*’ to a child’s hunger and satiety cues is identified as ‘responsive feeding’^(6)^. Adults, however, may ignore or misinterpret child cues and these practices fall within the domain of coercive control, for example pressuring the child to eat all the food on their plate, offering food in response to a child’s distress or using food ‘treats’ as reward for appropriate behavior. When parents use feeding practices that are not responsive to a child’s internal cues, it may teach children to eat for reasons other than hunger, disrupting self-regulation of energy intake with subsequent negative impacts on food preferences, dietary quality and long-term health consequences^(7)^.

The importance of the feeding practices used by educators in the Early Childhood Education and Care (ECEC) setting is increasingly being recognised. Use of practices by educators, that are consistent with autonomy support and structure, have been associated with higher intake of fruit and vegetables amongst children^(8,9)^. However, most of the literature describing the use of feeding practices by educators originates in the USA and Europe^(10)^ with very little known about the practices used by educators in Australian ECEC settings. This is despite almost one million Australian children under 5 years of age attending centre-based ECEC services, with 48 % of children attending by age 2 years^(11)^.

ECEC is a highly regulated sector in Australia. The workforce includes educators with a mix of qualifications, spanning from a 1-year Certificate III entry qualification to a 4-year teaching degree, with most educators holding a 2-year vocational Diploma of ECEC^(12)^. All ECEC services need to work within the National Quality Framework (NQF), which encompasses National Laws and Regulations and a quality rating system that drives continuous quality improvement^(13)^. Drawing on contemporary research, the NQF promotes child agency and autonomy supported by adult practices that are respectful of and responsive to individual child needs. This extends to pedagogical practices to support children’s healthy development and lifestyle. A companion document to the NQF is the ‘National Healthy Eating Guidelines and Physical Activity Recommendations for Early Childhood’, commonly referred to as the ‘Get Up & Grow’ guidelines^(14)^. These guidelines contain three recommendations related to feeding practices, which could all be considered to fall within the structure domain^(5)^ – that educators ‘*sit with children while they eat*’, ‘*eat and drink the same things as the children*’ and ‘*ensure that you are modelling healthy eating behaviours*’.

Two qualitative studies document barriers to the use of responsive feeding practices in the Australian ECEC setting^(15,16)^. In one study, educators reported being constrained by time and staffing, thereby having to establish a meal-time routine that met the ECEC centre’s needs, rather than being responsive to an individual child’s needs^(16)^. The second study was with pre-service educators (*n* 19) who reported a gap between knowledge about optimal feeding practices learned during training and what was observed when on practical placements^(15)^. Pre-service educators also noted that policies and procedures regarding mealtimes were not easily accessible to educators or parents. With many of a child’s meals consumed in ECEC during their parent’s working week, *how* children are fed by educators may have considerable influence on children’s developmental trajectory. Therefore, the aim of this study is to describe the use of feeding practices amongst a group of Australian educators, using both direct observation of practices and policy and self-report.

## Methods

A convenience sample of thirteen ECEC centres across Brisbane and the Sunshine Coast in Queensland, Australia were invited to participate in a study which aimed to develop and evaluate a professional development program to promote responsive feeding practices known as NOURISH: Early Childhood Education (NOURISH:ECE). All were centre-based ECEC services, providing care for children aged 6 weeks to 5 years of age from approximately 7 am to 6 pm Monday to Friday. All centres were operated by one large organisation which manages over 300 centre-based ECEC services across the state of Queensland, therefore access to the centres was first negotiated with this organisation. Centre Directors were then approached by research staff who explained the study procedures. Directors decided whether to participate in consultation with their staff. The findings presented here are the baseline self-report and direct-observation data collected as part of the NOURISH:ECE project between December 2018 and June 2019.

Ten ECEC centres agreed to take part in the study. Centres varied in size, catering for between 46 and 125 children/d. According to publicly available data from the Australian Early Development Census^(17)^ all centres were in areas with a higher proportion of children who are considered developmentally vulnerable in two of the five domains of the Australian Early Development Census compared with the National average of 11 %. The Australian Early Development Census is a national indicator of early childhood development collected every 3 years. Children commencing their first year of compulsory education are scored on five domains, physical health and well-being; social competence; emotional maturity; language and cognitive skills and communication skills and general knowledge. The proportion of children who are developmentally vulnerable on two or more of the five domains can indicate how well early childhood health and development is supported within a region^(18)^. Four centres were in areas with a higher proportion of children who were developmentally vulnerable on two domains (16·4 %, 17·8 %, 18·8 % and 21·4 %) compared with the Queensland state average of 13·9 %. Two of the three centres that declined participation were located in areas with fewer vulnerable children than the state or national averages.

After Centre Directors had agreed to take part, all individual educators (approximately 140 permanent or casual staff, working full-time or part-time) were invited to participate through the distribution of paper-based participant information sheets. Written informed consent was obtained to participate in the direct observation of mealtimes and/or an online survey. Of the 140 educators invited, 88 completed the online survey regarding feeding practices (response rate 63 %) with demographic data presented in Table 1. Ninety-five percent were female (*n* 84), and median duration working in ECEC was 12 years (interquartile range (IQR) = 5–20).

**Table 1:** Characteristics of educators providing self-reported data on feeding practices (*n* 88)

Variable	%	*n*
Age		
18–25 years	15	13
26–35 years	16	14
36–45 years	34	30
46–55 years	17	15
56–65 years	16	14
< 65 years	2	2
Gender		
Female	95	84
Education		
Any level of high school	1	1
Certificate III	25	22
Diploma	50	44
Bachelor’s degree	24	21
Own children		
Yes	71	62
Room at centre		
Babies	16	14
Toddlers	15	13
Pre-kindy	22	19
Kindergarten	28	25
Casual or floating staff	19	17
Employment status		
Full time	67	59
Part time	25	22
Casual	8	7

### Direct observation of feeding practices

Data were collected on provider practices and program policies using components of the ‘Environment and Policy Assessment and Observation’ tool (EPAO-2017)^(19)^. Slight modifications were made to the wording to suit the Australian ECEC context. The term educator was used instead of ‘provider’, and some food descriptions were altered e.g. crumbed instead of ‘breaded’, porridge instead of ‘grits’. One additional question was ‘Describe this meal?’ with the response options: progressive, standard or flexible. A progressive mealtime refers to an extended period in which the food is accessible to children and the decision about when and how long to engage in the mealtime is child-led. A standard mealtime refers to a set time frame where children are asked to finish playing/activities and come together for the meal or snack to be served. A flexible mealtime is one in which meal and snack times are approximate. Food can be provided earlier if children indicate that they are hungry or serving of food can be delayed if children are engaged in another activity.

Three research staff completed online training in the use of the EPAO-2017^(19)^ via the website of the University of North Carolina in the USA^(20)^. Resources include a user manual and certification videos, with staff completing their certification against the gold standard videos on the site.

Trained staff then visited each ECEC centre alone or in pairs, over 1 to 2 d (depending on the size of the centre) and completed the modified nutrition components of the EPAO-2017 at morning tea, lunch and afternoon tea in each room. In Australian ECEC centres this typically consists of a ‘Infant’ room catering for children aged 6 weeks to 15 months, ‘Toddlers’ from 15 months to 3 years, ‘Pre-kindergarten for children aged 3 to 4 years, and ‘Kindergarten’ which is for children in the year prior to starting formal schooling. Educators understood the purpose of the study to be that research staff wanted to describe what normally happens at mealtimes in ECEC. Direct observation occurred before the online survey, to avoid highlighting the specific focus on feeding practices. The date of each visit was pre-arranged with Centre Directors and research staff spent approximately 8 h/d at each centre, arriving about 1 h before the first meal of the day was served, which was always morning tea in the ‘Infant’ room. In the ‘Infant’ room, the observed mealtimes were those in which solid foods were served to groups of infants and young children. Instances in which educators bottle-fed individual children were not included in the observation.

Centre nutrition policies were examined using the checklist in the ‘Program policies’ section of the EPAO-2017. Any relevant sections of policy that described the use of feeding practices were extracted and are reported verbatim in the results.

### Self-report feeding practices

Self-reported feeding practices were collected via an online survey using the feeding-related items from the Childcare Food and Activity Practices Questionnaire (CFAPQ)^(21)^. The CFAPQ consists of 41 food-related items which form eight scales – Restriction (six items); Monitoring (four items); Modelling/Encourage balance and variety (seven items); Involvement/Environment (five items); Teaching about nutrition (three items); Pressure to eat (four items); Child control (five items); Emotion regulation/Food as reward (five items) and a single item ‘do you encourage the children to eat healthy foods before unhealthy ones?’. Items are measured on a five-point Likert scale ranging from disagree to agree, or never to always. Scale scores can range from one to five with a higher score representing greater use of that practice.

Three changes to wording were made to suit the Australian setting with ‘sweets’ changed to desserts, ‘cookies’ to biscuits, and ‘candy’ to lollies. The survey was managed using REDCap (Research Electronic Data Capture)^(22,23)^ hosted at the Queensland University of Technology and distributed to participants using their nominated email address.

### Data analysis

Data from paper copies of the EPAO-2017 were entered into an excel spreadsheet by a research assistant, checked by RB for accuracy then exported to IBM SPSS v25. The proportion of mealtimes per ECEC centre at which an educator was observed using each feeding practice at least once during the meal was calculated. This approach has been used in other studies^(24)^.

Online survey data were exported from REDCap to IBM SPSS v25 for analysis. Scores were calculated for the seven scales of the CFAPQ by averaging items within each scale. Internal consistency was acceptable for restriction, *α* = 0·72; monitoring, *α* = 0·91; modelling/encourage balance and variety, *α* = 0·77; involvement/environment *α* = 0·74 and teaching about nutrition, *α* = 0·70. To improve consistency, the item ‘I allow the children to help prepare meals’ was removed from the Involvement/Environment scale. As the remaining four items relate to the type of food provided, this scale was referred to as ‘Environment’. Similarly, the item ‘I tell the children what to eat and what not to eat without explanation’ was removed from the teaching scale. Internal consistency was fair for Pressure to eat, *α* = 0·61 and Child control, *α* = 0·61 (even with item ‘If the children don’t like the food that is being served, do you make something else? deleted to improve consistency) but was poor for Emotion regulation/Food as reward (*α* = 0·57). These three scales also showed low internal consistency in the original validation study, with *α* = 0·64, 0·54 and 0·56, respectively^(21)^.

## Results

### Direct observation of feeding practices and policy

A total of 120 meals were observed across the ten centres (morning tea, *n* 42; lunch *n* 41; afternoon tea, *n* 37). Nine centres provided food for the children. Of these, one had a kitchen onsite, while the remainder used a catering company. Meals were not provided for educators. As centres were all administered by the same ECEC organisation, all shared the same nutrition policy. This policy covered the topics of nutrition, mealtimes, preparing meals, food storage and safety and bottle feeding, and applied to centres that provide food and those where children bring food from home. There was no information regarding the type, quality or amount of food that should be provided to children, with readers referred to the Australian Dietary Guidelines^(25)^ and ‘Get Up and Grow’ guidelines^(14)^. Excerpts from the nutrition policy relating to feeding practices are noted in Table 2 and broadly describe how to enact responsive feeding practices across the three domains of autonomy support, structure and coercive control^(5)^.

**Table 2:** Feeding practices addressed in the childcare provider’s nutrition policy, and equivalent construct

Extracts from the childcare provider’s nutrition policy	Feeding construct
• Incorporate concepts regarding healthy food choices into the program. • Build children’s agency and autonomy by supporting them to choose what, when and how much they eat. • Encouraging children to eat healthy food without instructing them to eat food they do not like or to eat more than they want. • Engaging children in conversation about healthy food choices.	Autonomy support or promotion
• Model healthy eating habits when sharing mealtimes with children. • Sitting with and engaging children in conversations to create a relaxed and enjoyable mealtime atmosphere. • Being responsive to individual hunger needs by allowing children to eat outside routine mealtimes and feeding infants individually at different times. • Being patient with slow or “fussy” eaters.	Structure
• Never use food or drink to reward or punish children. • Do not withdraw food from children or make judgments about food provided by parents/guardians.	Coercive control

Of the 120 mealtimes observed, 39 (33 %) were standard, 59 (49 %) flexible and 22 (18 %) progressive. The progressive style of mealtime was most often implemented at morning and afternoon tea. Only three lunch mealtimes were classified as progressive and these were in rooms with older children (*n* 2 kindergarten, *n* 1 pre-kindergarten). The most common way in which food was served to children across mealtimes was ‘The educator served most foods and decided what size portions to give to the children’, which was observed at 76 meals (63 %). The next two most frequently observed styles of food provision were used far less often – ‘Children served themselves most/all foods and decided what size portions to take’ at only 18 meals (15 %) and ‘Children brought food from home’ at 14 meals (12 %).

The median proportion of mealtimes per centre at which educators were observed to use an authoritative feeding style was high, at 78·9 % (IQR = 66·7–91·7). An authoritative feeding style is defined in the EPAO-2017 as a balance between encouraging children to eat healthy foods and allowing children to make their own food choices, as well as using reason and education, rather than bribes or threats^(19)^. This is consistent with the low proportion of meals at which use of reward or pressure to eat were seen – the proportion of meals per centre at which educators were observed to use 13 different feeding practices is shown in Table 3. While educators at each centre frequently sat with children and discussed the foods they were eating (median of 75 % and 71·5 % of meals respectively), occurrence of enthusiastic role modelling (22·2 %), eating the same foods as children (19·6 %) or being seen to eat fruit or vegetables (3·3 %) was much less. Of note, in two centres, no educators were observed eating the same food as children at any meal. Across all ten centres, no educators were observed eating a ‘complete’ meal with children, i.e. modelling eating their own meal from hunger to satiety. The number of occasions on which educators were observed to eat unhealthy foods (classified in the EPAO-2017 as fast food, sweet snack, salty snack or sweetened beverage^(19)^) was so infrequent, that data are not presented in the table – sweet snack, *n* 2 mealtimes; sweetened beverage, *n* 1.

**Table 3:** Proportion of mealtimes (*n* 120 meals) per ECEC centre (*n* 10) at which an educator was observed using each feeding practice at least once during the meal

Practice	%, Median	IQR	Feeding construct
Did the educator eat any of the following foods in front of the children?			
The educator ate the same foods as the children	19·6	5·8–33·3	Structure
The educator ate fruits or vegetables	3·3	0–14·9	
How often were the following interactions observed between the educator and the children?			
The educator sat with the children	75·0	70·2–83·7	Structure
The educator enthusiastically role modelled eating healthy foods	22·2	0–33·3	
The educator encouraged children to try the foods on their plates			
Mean	63·6		Autonomy support or promotion
sd	18·2		
The educator praised a child for eating healthy foods	12·7	5·8–19·2	
The educator talked with the children about the foods they were eating			
Mean	71·5		
sd	9·33		
How often did the educator support or hinder children’s self-regulation?			
The educator pressured a child to eat	19·9	12·5–27·1	Coercive control
The educator required the child sit at the table until he/she cleaned their plate.	0	0–0	
How often did the educator use rewards or bribes?			
The educator promised something other than food for eating	0	0–6·8	Coercive control
The educator used food as a reward or bribe for eating a less preferred food	0	0–11·8	
The educator used food as a reward or withheld food as a punishment for behavior	0	0–0	
The educator used food to calm an upset child	0	0–7·1	

### Self-report feeding practices

Scores for feeding practice scales (median and IQR) are reported in Table 4. Educators reported high levels of modelling of healthy eating (median score of 4·9, out of a possible 5, IQR = 4·4–5·0) and low levels of pressure to eat (median = 2·0, IQR = 1·3–2·5). Scores on the ‘environment’ and ‘teaching’ scales were also high, both with a median score of 4·5 (IQR = 3·5–5·0 and 4·0–5·0, respectively), indicating that educators felt most foods provided at their centre were healthy and they discussed healthy eating with the children.

**Table 4:** Self-reported feeding practices of educators (*n* 88) measured using the childcare food & activity practices questionnaire (CFAPQ)^(21)^

Scale	Score* – median	IQR	Feeding construct
Teaching†,‡	4·5	4·0–5·0	Autonomy support or promotion
Encourage healthy foods§,‖	4·0	4·0–5·0	
Monitoring§	3·8	2·6–4·5	Structure
Modelling†	4·9	4·4–5·0	
Environment†	4·5	3·5–5·0	
Child control§	3·5	3·0–3·8	
Restriction†	3·0	2·5–3·5	Coercive control
Pressure to eat†	2·0	1·3–2·5	

* Possible score of 1–5.

† Items measured on five-point Likert scale: disagree, slightly disagree, neutral, slightly agree, agree.

‡ *n* 87.

§ Items measured on five-point Likert scale: never, rarely, sometimes, mostly, always.

‖ Single item ‘do you encourage the children to eat healthy foods before unhealthy ones?’.

## Discussion

With almost one million Australian children attending centre based ECEC services each day^(11)^, educators have an important role in supporting children’s health and well-being through the creation of optimal mealtime environments. This study is one of the first to describe the feeding practices used by educators in Australian ECEC settings, using both direct observation and self-report. The core practice of responsiveness was embedded within the ECEC organisation’s nutrition policy, and overall, educators reported, and were observed to use, practices that are consistent with ‘responsive feeding’^(6)^. The mealtime environment within these centres reflects the intent of Australia’s NQF^(13)^, which promotes child agency and autonomy and the need for pedagogical practices that are responsive to individual children.

While most mealtimes were adult-led, and the ‘educator served most foods and decided what size portions to give to the children’, children were largely able to decide which of these foods to eat and how much to consume. Practices that fit within the domain of coercive control^(5)^ were rarely observed, such as pressuring a child to eat more, or using food as a reward or bribe. This was consistent with the low levels of pressure on children to eat reported by educators. However, educators also reported often encouraging the children to eat healthy foods before unhealthy ones, with the item having a median score of 4·0 (IQR = 4·0–5·0). Some health practitioners have expressed concern about whether this type encouragement could be more consistent with pressure to eat or reward for eating, depending on the intent of the adult using the practice^(26)^.

Consistent with the NQF^(13)^, centre policy supported the use of practices which were aligned with autonomy support and appropriate levels of structure^(5)^. The organisational policy included statements such as ‘build children’s agency and autonomy by supporting them to choose what, when and how much they eat’ and encouraging educators to sit with and engage children in conversations, particularly around healthy food choices. These practices were frequently observed by the research team – educators at each centre frequently sat with children and discussed the foods they were eating (median of 75 % and 71·5 % of meals respectively), and educators self-reported high levels of agreement with the ‘teaching’ scale of the CFAPQ^(21)^, in which items relate to discussing the health and nutritional value of foods with children. Scores on the CFAPQ ‘environment’ scale were also high, indicating that educators felt the majority of foods provided at their centre were healthy. This may explain why scores for ‘restriction’ and ‘monitoring’ were 3·0 and 3·5 respectively on the 1–5 scale. Due to the presence of an overall healthy food environment, educators were generally neutral about the need to guide or regulate children’s intake of desserts or biscuits, and only sometimes needed to monitor the intake of high-fat foods or sugary drinks. However, there is a need for further research to examine the use of ‘restriction’ and ‘monitoring’ (and feeding practices more broadly) in centres where children bring meals from home in lunchboxes. There is no published data on the number of ECEC centres in Australia that provide food *v*. those in which children bring lunchboxes. In a study of 17 Australian ECEC services in which the contents of 355 children’s lunchboxes were examined, less than 1 % of lunchboxes met setting-specific nutrition guidelines and over half contained discretionary foods^(27)^. It is plausible that educators may use ‘restriction’, ‘monitoring’ and ‘encouraging the children to eat healthy foods before unhealthy ones’ if they perceive that lunchbox contents are not consistent with healthy eating. Another study by the same research group^(28)^, examined educator practices in twenty-two ECEC lunchbox centres (*n* 448 children), by using twenty-one items from the EPAO-2017 to create a composite score representing ‘use of feeding practices that support children’s healthy eating’. The mean score was 1·86 (sd = 0·22) on a scale of 1–3, but the frequency of use of specific practices was not reported.

Imitation of other people’s behavior by children is a powerful way to facilitate learning (i.e. observational learning^(1)^), and it is thought this can be harnessed to promote food acceptance, particularly in relation to vegetables^(29)^. Role modelling by educators is acknowledged as an essential component of multi-level interventions to promote healthy eating amongst young children in ECEC^(10,30)^ and specific guidance regarding modelling is incorporated into the ‘Get Up and Grow’ practice guidelines for educators^(14)^. One area in the NOURISH:ECE study in which there appeared to be a discrepancy between centre policy, self-reported and observed feeding practices, was role modelling. Centre policy stated, ‘model healthy eating habits when sharing mealtimes with children’ and levels of self-reported modelling were very high, with a median score of 4·9 (IQR 4·4–5·0). However, the direct observation of practices indicated that enthusiastic role modelling occurred at only approximately one in every five meals observed at each ECEC centre (22·2 %). Similarly, as these centres did not make provision for educator meals, educators were not often observed eating the same foods as children (median of 19·6 % meals per centre) and rarely seen eating fruits or vegetables (3·3 % of meals per centre). These findings contrast with those in other countries. Direct observation of practices in nine ECEC centres in the Netherlands using the EPAO-2017 revealed high levels of role modelling^(24)^. Of 135 eating occasions observed (which included morning tea, lunch and afternoon tea), educators ate with children at 76·9 % meals and ate the same food at 50 %. In the USA, researchers using the Meal-time Observation in Childcare tool (*n* 10 centres)^(31)^ observed educators enthusiastically role model healthy eating at 58 % of meals and consuming fruit or vegetables at 24 % and 39 % of eating occasions. However, both studies found more frequent use of pressure to eat by educators, namely ‘times the child was pressured or encouraged to eat more’ at 52·3 % of meals^(24)^ and ‘pressured child to eat when they refused’ at 88 %^(31)^ compared with 19·9 % of eating occasions in the current sample.

International discrepancies could be due to many reasons – differences in measurement, cultural differences in the application of feeding practices, or differences in training and policy governing educators in different jurisdictions. Two qualitative studies of feeding practices in Australian ECEC settings, educators and trainees report that rigid centre policies prevent the use of responsive feeding in practice^(15,16)^. This is unlikely to be the case in the current study given that the ECEC operator’s policy explicitly supports modelling, but there may be other workplace, professional and personal barriers that prevent educators from modelling, regardless of how much they value this practice. For example, key educators need to take breaks at scheduled times, often leaving casual staff to cover meals. The opportunity for role modelling by familiar and trusted adults is potentially lost. Malek-Lasater *et al.* also note the discrepancy in the promotion of optimal mealtime practices between health and education disciplines^(31)^ arguing that ‘*the use of responsive feeding practices needs to be incorporated into quality measurements and teaching practices endorsed by education-related entities in order to ensure they are understood and implemented in the class-room*’.

Food service also plays an important role. Meals were not provided to educators at the centres participating in this study and educators may be reluctant to consume any of the food that is provided for children. The style of food service has implication for how meals are served to children. A centre that prepares food in bulk to provide meals to a large number of children, may be less able to implement a progressive style of meal, compared with a centre in which children bring lunch boxes from home. Anecdotally, the use of progressive meals is increasing. The idea that children are solely responsible for deciding when and how long to engage in mealtimes according to their internal cues of hunger and satiety, is consistent with supporting a child’s self-regulation of energy intake. However, the burden on educators to safely implement this in rooms with infants and toddlers is likely to be significant, and perhaps why progressive meals were rarely used at lunchtime in the current study, and only with older children who are largely independent with self-feeding. The use of progressive meals represents less ‘structure’^(5)^ which may also impact the atmosphere of meals, with fewer opportunities for discussion amongst groups of children, and role modelling by educators and peers. Trade-offs might be required to balance the use of progressive meals with the social goal of eating together as an ECEC community^(32)^. Exploring the differential use of structure, autonomy supportive and controlling practices across the age and developmental groupings are typically seen in ECEC – infants, toddlers, pre-kindergarten and kindergarten – is an important premise for future research which can inform training and education programs for educators.

Other opportunity costs such as providing meals to staff to facilitate role modelling of healthy food intake could also be considered. Economic evaluation methods such as cost-effectiveness modelling can inform decisions about implementation of responsive feeding policies and quantify the trade-offs associated with different approaches. The characteristics of centres where responsive feeding practices are successfully implemented could be compared with centres where responsive practices less frequently, thereby identifying potential avenues for interventions to promote and support optimal feeding environments.

### Strengths and limitations

The direct observation of feeding practices is a strength of this study, as it overcomes the reporting bias associated with self-report practices. Social desirability bias may explain the high level of role modelling reported in this sample, and the discrepancy between observed and self-reported practices. However, the limitations of this study mean that this apparent discrepancy must be interpreted with caution. Practices were observed with the ‘meal’ as the unit of interest and reported by centre. There was no direct comparison of an individual’s self-reported level of a given feeding practice with the observation of that educator’s behaviour. As educators could choose to participate in one or both activities, the sample for self-report *v*. observed data would be slightly different.

There are also limitations regarding the measurement tools used. It is not known whether educators are conceptualising practices in the same way as intended in the questionnaire items. There is also an absence of tools to assess feeding practices in the Australian ECEC setting with adequate evidence of validity and reliability. In the interim, this study provides insight into the applicability of existing validated tools that may be used to assess educator practices in Australia.

There are also limitations regarding the use of EPAO-2017^(19)^. New items were recently developed and tested in family day-care homes, to capture educator feeding practices more comprehensively across the domains of autonomy support, structure and coercive control^(33)^. The EPAO-2017 also does not consider the frequency with which a practice occurs during an eating occasion. A practice need only occur once at a meal time to score ‘yes’ on an individual item. Future studies which examine the impact of educator practices on child outcomes in Australia could consider using tools that assess the intensity of a given feeding practice. One such example is ‘Table Talk’ which characterises verbal interactions at meal times^(34)^.

All centres were operated by one large organisation, and all but one provided food, which may limit variability in the types of practices seen. Investigation of feeding practices used in diverse types of centres across all Australian states and territories, is an area for future research e.g. private for-profit centres *v*. private not for profit, large providers *v*. small single service operators; as well as exploring educator practices in services in which children bring food to the centre in lunchboxes.

## Conclusions

This is the first study to quantitatively assess the feeding practices used by educators in Australian ECEC centres using both direct observation and self-report. Centre polices supported the use of responsive feeding practices, and this was reflected in the high frequency with which children could decide what and how much to eat, across both observed and self-report data as well as low levels of pressure to eat and use of food as a reward. The only apparent discrepancy was in regard to role modelling. Despite policy support, and high levels of educator self-report, frequency of observed role modelling was low. Further research addressing how educators conceptualise feeding practices, as well as how and under what circumstances they are used, may shed light on why this important aspect of mealtimes is rarely implemented in practice.
